# Supplementary figures and images for: A noncanonical role for dynamin-1 in regulating early stages of clathrin-mediated endocytosis in non-neuronal cells
Source: PLoS Biol. 2018 Apr 18;16(4):e2005377. doi: 10.1371/journal.pbio.2005377 (PMC5927468; doi:10.1371/journal.pbio.2005377)

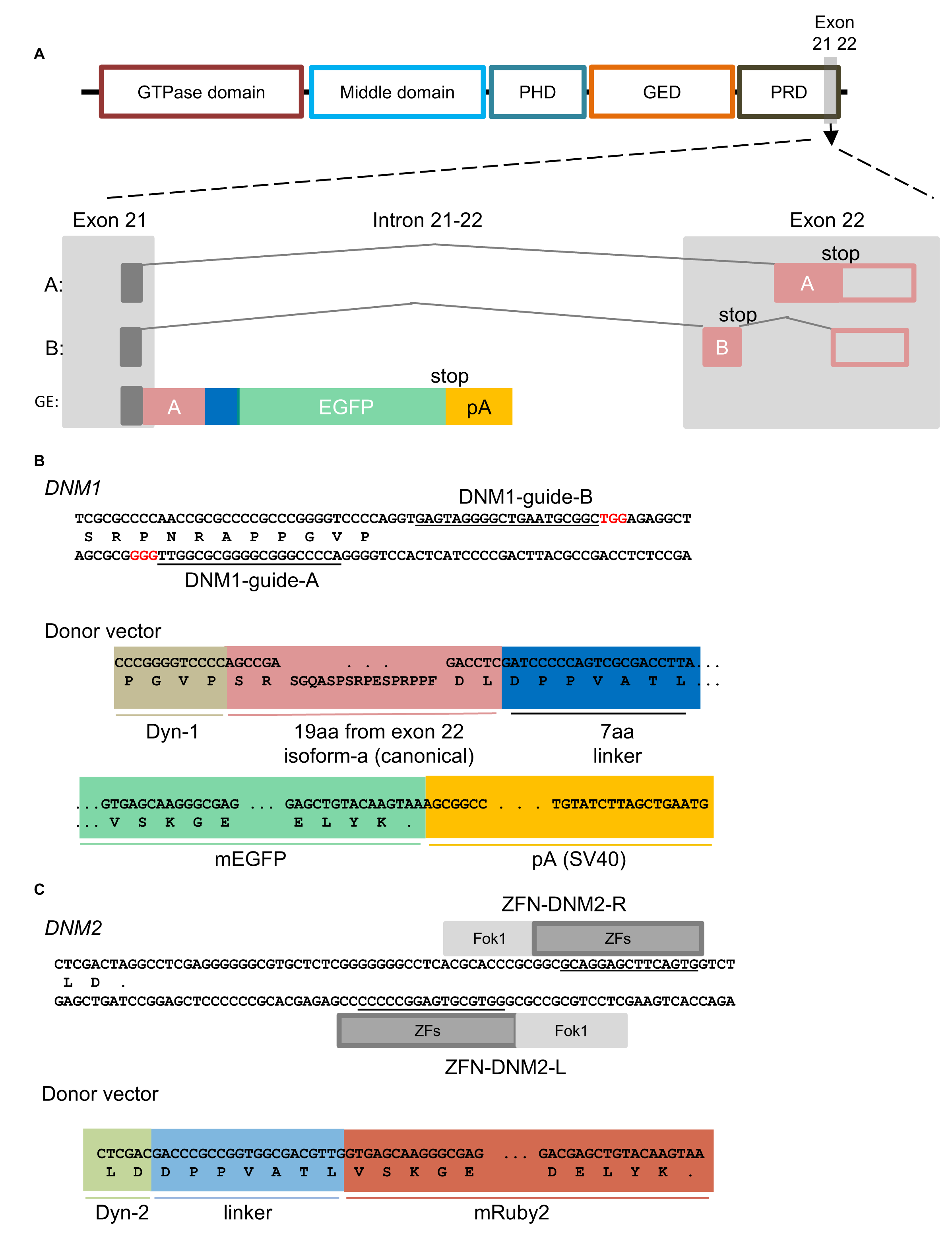

Supplement: S1 Fig — (A) Domain and genomic structure of DNM1 illustrating the C-terminal splice variants to illustrate splice variants A and B derived from exons 21 and 22 (filled red box) and 3′ UTR (open red box). To express the Dyn-1-EGFP fusion protein, the last 19 amino acids from splice variant A from exon 22 were introduced in frame in exon 21 (dark grey), followed by a 7-amino acid linker (blue), EGFP (green), and SV40 poly adenylation signal (orange). (B) Design of sgRNA guide A and B which targets the splice region in exon 21. The guide targeting sequences (underlined) and PAM sequences (red) are shown. For the donor vector, the DNA and amino acid sequences are shown for the junctions between exon 21, the inserted Dyn-1 C-term, the linker, EGFP and the poly adenylation signal. The color code is as in panel A. (C) Approach used for ZFN-mediated genome-editing of DNM2, as previously described [32,34] and the expected amino acid sequence for the Dyn2-mRuby2 fusion protein. Dyn1, dynamin-1; sgRNA, single-guide RNA; ZFN, Zinc Finger Nuclease. (TIF) [file pbio.2005377.s001.tif]

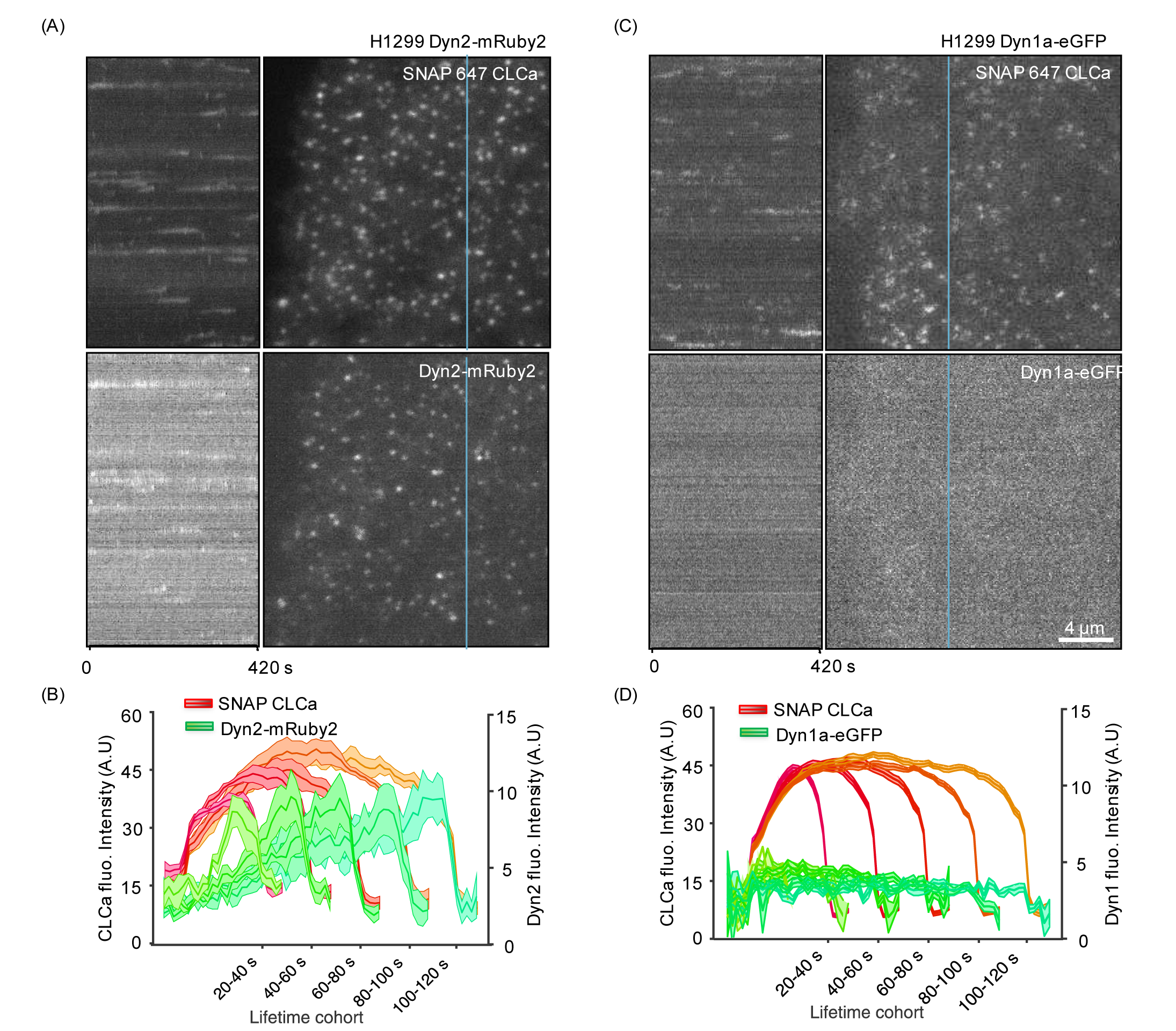

Supplement: S2 Fig — (A, C) Representative TIRF image and corresponding kymograph of dynamic SNAP(647)-CLCa-labeled CCPs and Dyn2-mRubyend (A) or Dyn1a-eGFPend (B) in genome-edited H1299 cells. (B, D) Corresponding quantification of the averaged intensities of CLCa and Dyn2-mRubyend (B) or Dyn1a-eGFPend (D) recruitment for the indicated lifetime cohorts. Data from 6,647 CCPs from 5 independent movies, containing a total of 15 cells (B) and data from 74,805 CCPs from 10 independent movies, containing a total of 29 cells (D). CCP, clathrin-coated pit; CLCa, clathrin light chain a; Dyn1, dynamin-1. (TIF) [file pbio.2005377.s002.tif]

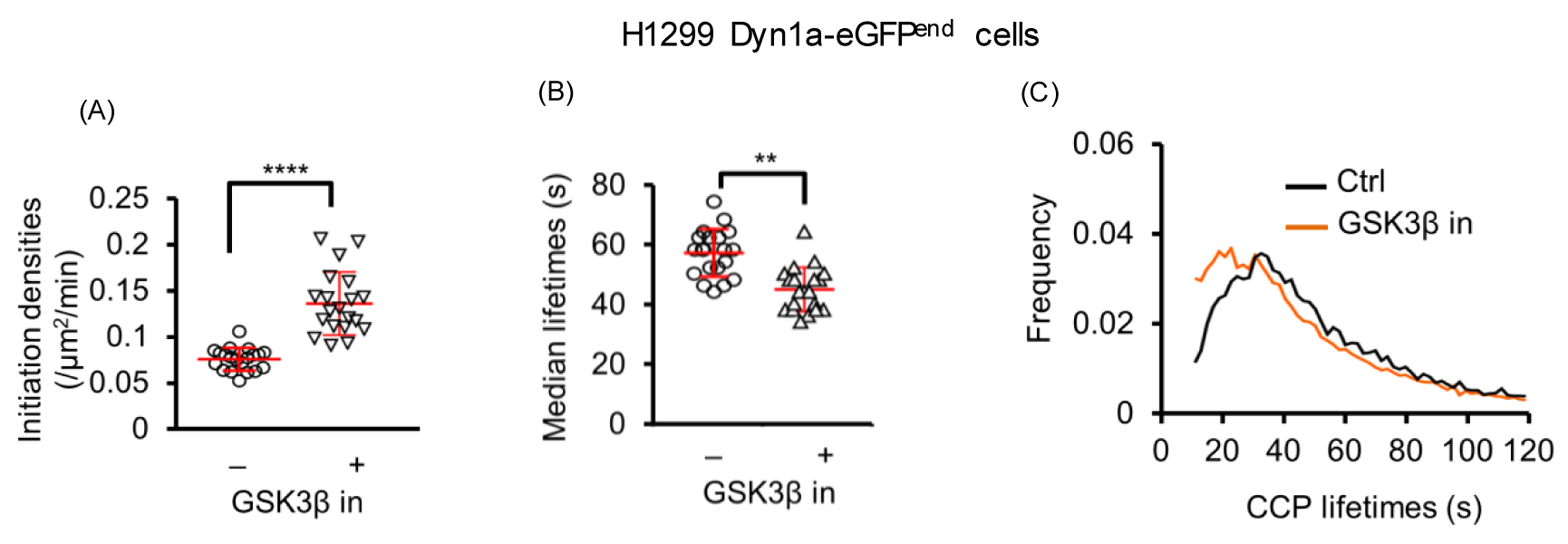

Supplement: S3 Fig — (A) CCP initiation rates, (B) CCP lifetimes, and (C) lifetime distributions of all CCPs in H1299 cells genome edited to express endogenously tagged Dyn1a-eGFP. Each point represents the value derived from a single movie, with 2–4 cells/movie. (** p ≤ 0.01, **** p ≤ 0.0001). The underlying data of panels A and B can be found in S1 Data. CCP, clathrin-coated pit; Dyn1, dynamin-1. (TIF) [file pbio.2005377.s003.tif]

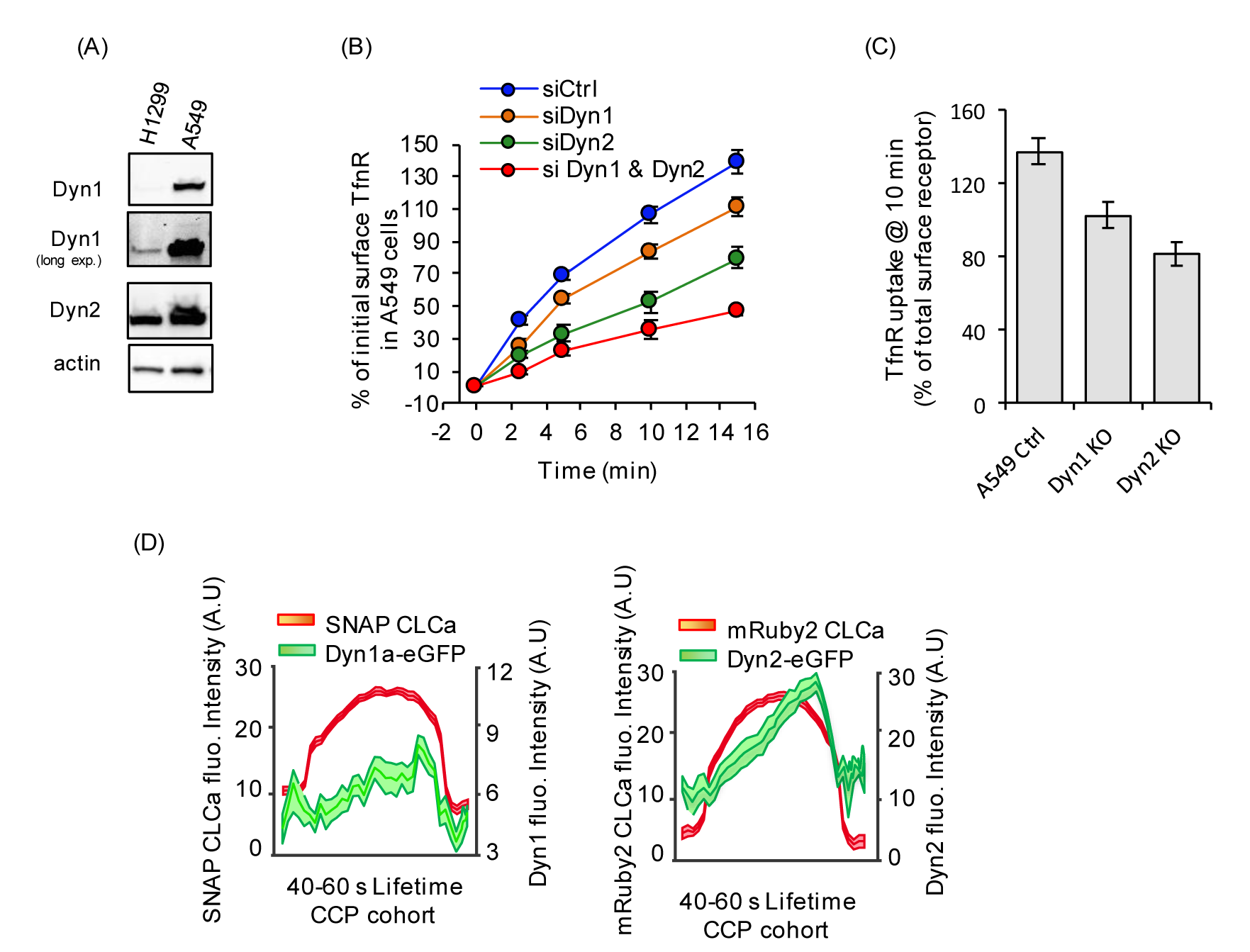

Supplement: S4 Fig — (A) Differential expression of Dyn1 versus Dyn2 in H1299 versus A549 cells. In H1299 cells, Dyn2 is expressed at approximately 6-fold higher levels than Dyn1. In A549 cells, Dyn1 is expressed at approximately 5-fold higher levels than Dyn2 [39]. (B) TfnR endocytosis in parental A549 cells treated with the indicated siRNAs. (C) TfnR uptake at 10 min in parental, Dyn1KO, and Dyn2KO A549 cells. (D) Quantification of the average recruitment of Dyn1a-eGFP or Dyn2-eGFP to CCPs with lifetimes between 40 and 60 s (4,420 CCPs positive for Dyn1 and 3,961 CCPs positive for Dyn2 were identified and analyzed from 11 movies containing 2–4 cells per movie), as in Fig 5E; however, the Dyn1a-eGFP data is rescaled to illustrate that Dyn1, like Dyn2, peaks at late stages of CME in these cells. The underlying data of panels B and C can be found in S1 Data. CCP, clathrin-coated pit; CME, clathrin-mediated endocytosis; Dyn1, dynamin-1; siRNA, small interfering RNA; TfnR, transferrin receptor. (TIF) [file pbio.2005377.s004.tif]

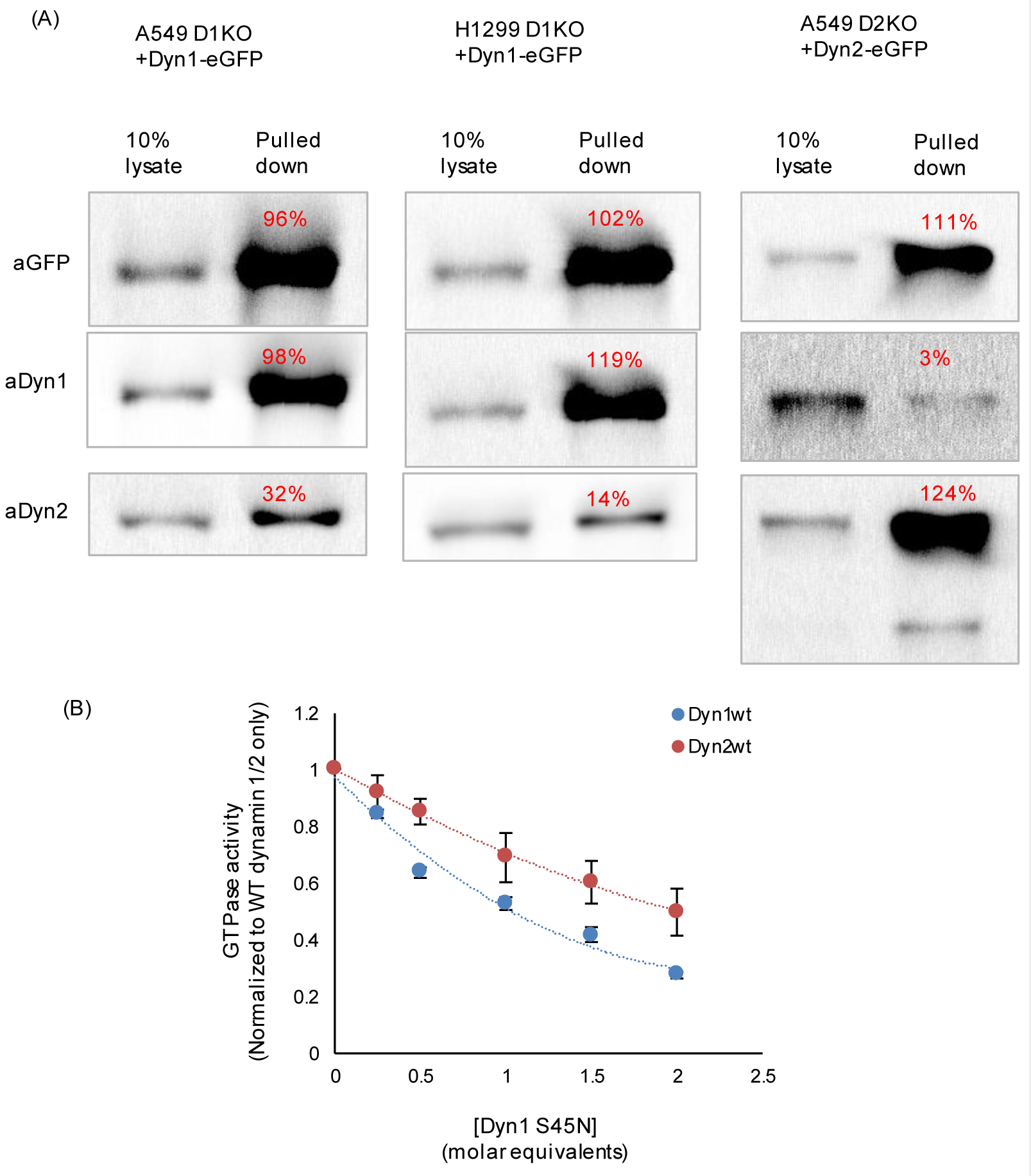

Supplement: S5 Fig — (A) Western blots and quantification (red) of bands showing extent of pulldown of Dyn1-eGFP or Dyn2-eGFP using anti-eGFP nAb-beads and coimmunoprecipitation of the other isoform. Data are representative of 3 independent experiments. (B) The inhibition of assembly stimulated GTPase activity of Dyn1 (blue) or Dyn2 (red) in the presence of increasing concentrations of GTPase-defective Dyn1S45N, which will inhibit assembly-stimulated GTPase activity by co-assembling with WT-dynamin on lipid nanotube templates. The underlying data of panel B can be found in S1 Data. Dyn1, dynamin-1; GTPase, Guanosine Triphosphate hydrolase; WT, wild-type. (TIF) [file pbio.2005377.s005.tif]
