# Supplementary material for: A noncanonical role for dynamin-1 in regulating early stages of clathrin-mediated endocytosis in non-neuronal cells
Source: PLoS Biol. 2018 Apr 18;16(4):e2005377. doi: 10.1371/journal.pbio.2005377 (PMC5927468; doi:10.1371/journal.pbio.2005377)
Supplement: S1 Table — (DOCX) [file pbio.2005377.s009.docx]

**S1_Table:** Oligonucleotides used for genome-editing, mutagenesis and fusion constructs

| **nr** | **name** | **sequence** |
| --- | --- | --- |
| 1 | DNM1-LH-f | **GCGTAATACGACTCACTATAGGGCGAATTGGGTACCCACAGTCCCAGCAGG** |
| 2 | DNM1-LH-r | **GCCTGACCCGATCGGCTGGGGACCCCGGGCGGGGCGCGGTTG** |
| 3 | DNM1-C-assembly-f | **CCCGGGGTCCCCAGCCGATCGGGTCAGGCAAGTCCATCCCGTCCTGAGAGCCCCAGGCCCCCCTTC** |
| 4 | DNM1-C-assembly-r | **CTCGCCCTTGCTCACTAAGGTCGCGACTGGGGGATCGAGGTCGAAGGGGGGCCTGGGGCTCTCAG** |
| 5 | DNM1-Cterm-f | **CCCGGGGTCCCCAGCCGATCGGGTCAGGC** |
| 6 | DNM1-Cterm-r | **CTCGCCCTTGCTCACTAAGGTCGCGACTGGGGGATCGAGGTCGAAGGGGGGCCTG** |
| 7 | DNM1-eGFP-f | **CCCCCCTTCGACCTCGATCCCCCAGTCGCGACCTTAGTGAGCAAGGGCGAG** |
| 8 | DNM1-eGFP-r | **CTAGAGTCGCGGCCGCTTTACTTGTACAGCTC** |
| 9 | DNM1-pA-f | **GAGCTGTACAAGTAAAGCGGCCGCGACTCTAG** |
| 10 | DNM1-pA-r | **GGAACAAAAGCTGGAGCTCCACCGCGGTGGCGGCCGCCTGCTGTGTTCTTCC** |
| 11 | DNM1-RH-f | **CATCAATGTATCTTAGCTGAATGCGGCTGG** |
| 12 | DNM1-RH-r | **GGAACAAAAGCTGGAGCTCCACCGCGGTGGCGGCCGCCTGCTGTGTTCTTCC** |
| 13 | DNM2-LH-f | **GCGCGCGTAATACGACTCACTATAGGGCGAATTGGGTACCGCCCCGCCTCAGATCCC** |
| 14 | DNM2-LH-r | **CTTCGCCCTTAGACACCAACGTCGCCACCGGCGGGTCGTCGAGCAGGGATGGCTC** |
| 15 | DNM2-mRuby2-f | **GAGCCATCCCTGCTCGACGACCCGCCGGTGGCGACGTTGGTGTCTAAGGGCGAAG** |
| 16 | DNM2-mRuby2-r | **CACGCCCCCCTCGAGGCTTACCCTCCGCCCAGGCCGGCGAAC** |
| 17 | DNM2-RH-f | **GTTCGCCGGCCTGGGCGGAGGGTAAGCCTCGAGGGGGGCGTG** |
| 18 | DNM2-RH-r | **AAGGGAACAAAAGCTGGAGCTCCACCGCGGTGGCGGCCGCGGCCGCCCTGGGCCCAC** |
| 19 | DNM1-Nuclease-A-f | **CACCGACCCCGGGCGGGGCGCGGTT** |
| 20 | DNM1-Nuclease-A-r | **AAACAACCGCGCCCCGCCCGGGGTC** |
| 21 | DNM1-Nuclease-B-f | **CACCGGAGTAGGGGCTGAATGCGGC** |
| 22 | DNM1-Nuclease-B-r | **AAACGCCGCATTCAGCCCCTACTCC** |
| 23 | DNM1-S774A_mut_f | **CCGGACGCAGGGCGCCCACGTCC** |
| 24 | DNM1-S774A_mut_r | **GGACGTGGGCGCCCTGCGTCCGG** |
| 25 | DNM1-S778A_mut_f | **CGCCCACGTCCGCCCCCACGCCGC** |
| 26 | DNM1-S778A_mut_r | **GCGGCGTGGGGGCGGACGTGGGCG** |
| 27 | SNAP-f | **AATACGACTCACTATAGGGCGAATTGGGTACCGAATTCTGCCACCATGGACAAAGACTGC** |
| 28 | SNAP-r | **GATCCAGCTCAGCCATGCTGCCTCCTGAACCTCCACCCAGCCCAGGCTTGC** |
| 29 | SNAP-CLCa-f | **GCAAGCCTGGGCTGGGTGGAGGTTCAGGAGGCAGCATGGCTGAGCTGGATC** |
| 30 | CLCa-r | **GGGAACAAAAGCTGGAGCTCCACCGCGGTGGCGGCCGCGTCGACTTAGTGCACCAGCGG** |
| 31 | mRuby2-f | **AATACGACTCACTATAGGGCGAATTGGGTACCGAATTCTGCCACCATGGTGTCTAAGGGC** |
| 32 | mRuby2-r | **GATCCAGCTCAGCCATGCTGCCTCCTGAACCTCCCTTGTACAGCTCGTC** |
| 33 | mRuby2-CLCa-f | **GACGAGCTGTACAAGGGAGGTTCAGGAGGCAGCATGGCTGAGCTGGATC** |

Primers used for the assembly of the donor vectors for DNM1-EGFP (nr1-12), DNM2-mRuby2 (nr13-18), the nickase CRISPR/Cas9 guide RNA for DNM1 (nr19-22), DNM1 S774/8A mutagenesis (nr23 & 24) and for generation of the SNAP-CLCa and mRuby2-CLCa vectors (nr25-31). The donor DNA for DNM1-eGFP was assembled into the vector from five fragments: i) left homology (LH) arm, ii) DNM1 C-terminal 19 amino acids together with a seven amino acid linker (C-assembly), iii) monomeric eGFP (GFP), iv) SV40 polyadenylation signal (pA) and v) the right homology arm (RH), which were amplified with forward (f) and reverse (r) primers, as indicated. Likewise, the donor DNA for DNM2-mRuby2 was assembled into the vector from three fragments: i) the left homology arm (LH), ii) mRuby2 and iii) the right homology arm (RH). Primers 19-20 were used to generate the guides A and B for the nickase CRISPR-CAS9 guide RNA targeting DNM1. Primers 25-31 were used to generate CLCa-SNAP tagged lentiviral vectors.
